# Social Determinants of Health Impacting Access to Renal Dialysis for Racial/Ethnic Minorities

**DOI:** 10.7759/cureus.45826

**Published:** 2023-09-23

**Authors:** Joseph L Mercen, Kiely M Curran, Markeeta T Belmar, Jaron Sanchez, Ibrahim Hasan, Sahib Kalra, Parth M Raina, Sahil Patel, Dania Arrechavaleta, Vincent Lee, Paula Anderson

**Affiliations:** 1 Dr. Kiran C. Patel College of Osteopathic Medicine, Nova Southeastern University, Clearwater, USA; 2 Dr. Kiran C. Patel College of Osteopathic Medicine, Nova Southeastern University, Fort Lauderdale , USA; 3 Dr. Kiran C. Patel College of Osteopathic Medicine, Nova Southeastern University, Clearwater , USA; 4 Dr. Kiran C. Patel College of Osteopathic Medicine, Nova Southeastern University, Fort Lauderdale, USA

**Keywords:** scoping review, minority, ethnic, racial, chronic kidney disease, social determinants of health

## Abstract

Although widespread, the burden of disease presented by chronic kidney disease (CKD) is not equally distributed among all demographics. Examining the social determinants of health (SDOH) that relate to barriers to renal dialysis care in CKD can help to prevent future disparities. There has not been a study addressing the social factors that create barriers to care for ethnic minority patients with CKD. The aim of this scoping review is to address the SDOH that affects access to renal dialysis for ethnic minority patients in the United States. This study was based on the protocol published by the Joanna Briggs Institute. A total of 349 studies were identified from PubMed, EBSCOhost, and Embase. Each article was screened against population, concept, and context criteria in order to be considered for inclusion. The population was determined to be adults of all genders from underrepresented minority populations. The selected concept was SDOH. The context of this study was the United States population. From the articles selected by the search criteria, neighborhood of residence, mental health care access, glomerular filtration rate (GFR) methodology, socioeconomic status (SES), language barriers, immigration status, and military rank were identified as SDOH affecting access to renal dialysis care. While this study identified four social determinants, more research is needed for the investigation of other possible SDOH contributing to disparities related to CKD and access to renal dialysis care.

## Introduction and background

Chronic kidney disease (CKD) impacts millions of people worldwide and is growing in prevalence. In the United States, 800,000 end-stage renal disease (ESRD) patients require regular kidney dialysis treatment [1,2]. CKD is a cause of morbidity and mortality, as well as a contributor to rising healthcare costs [3]. The global mortality rate of CKD has increased by over 40% since the 1990s [1,4]. Nationwide, kidney disease creates millions of dollars in healthcare costs, and the economic burden continues to rise [5]. Social determinants of health (SDOH) may contribute to disparities in CKD. The United States Centers for Disease Control and Prevention (CDC) defines SDOH as “the conditions in the environments where people are born, live, learn, work, play, worship, and age that affect a wide range of health, functioning, and quality-of-life outcomes and risks” [6]. By examining the SDOH that relates to barriers to care in CKD, we can help prevent future disparities.

Factors identified in the background review of literature that may affect care in CKD may include socioeconomic status (SES) [2,7,8,9], membership in vulnerable populations (including prison inmates, homeless individuals, and undocumented immigrants) [4,10,11], race and sex [12-16], barriers created by the coronavirus disease 2019 (COVID-19) pandemic [17-19], insurance coverage [20], relationship with primary care provider (PCP) [21], transportation access [20,22,23], and utilization of at-home dialysis [24].

There has not yet been a scoping review addressing the SDOH that affects access to renal dialysis for ethnic minority patients. The aim of this scoping review is to identify existing literature on the different SDOHs that create barriers for ethnic minorities with CKD in receiving hemodialysis care. 

The findings of this study were earlier presented at the Nova Southeastern University Office of Graduate Medical Education Spring 2023 poster competition.

## Review

Methods

Eligibility Criteria 

**Figure 1:**
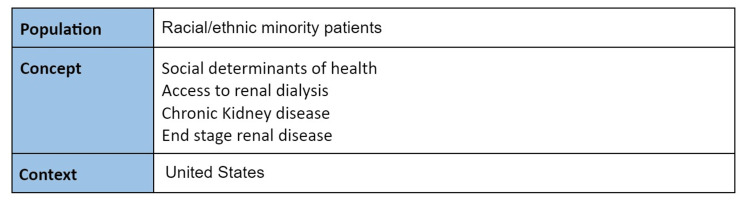
PCC framework organization of the inclusion criteria for this study. PCC: Population, Concept, Context

To be included in the scoping review, research publications had to explore the social factors affecting access to renal dialysis treatment in racial and ethnic minority patients with ESRD. The inclusion criteria were determined using a Population, Concept, Context (PCC) framework (Figure 1). Peer-reviewed journal publications were used if they were published between 2012-2022 and written in English. The research studies needed to look specifically at patients living and receiving care in the United States. The decision was made to limit the analysis to studies in the United States because healthcare and SDOH differ greatly throughout the countries of the world. Papers discussing clinician biases when dealing with ESRD were included.* *

Information Sources

An initial draft literature search was conducted on August 5, 2022, that yielded 349 articles. The last comprehensive literature search was executed on October 9, 2022. All information was sourced from online resources in databases. Only peer-reviewed published articles were included in the study.

Search Strategy

A systematic search was carried out following Preferred Reporting Items for Systematic Reviews and Meta-Analyses (PRISMA) guidelines on Embase (Excerpta Medica Database), PubMed, and CINAHL (Cumulative Index to Nursing and Allied Health Literature)(EBSCOhost) for all reviews published from 2013 to 2022 using a combination of keywords and medical subject headings (MeSH) related to disparities in healthcare, hemodialysis, ESRD, ethnic minority, United States of America, and SDOH. Additionally, one gray literature source (Google Scholar) identified no new articles. Boolean operators AND and OR were used to produce more focused and productive results. The search strategy was initially conducted in PubMed and later adapted to other databases to identify articles from the four databases. Studies that were included addressed physical, informational, and interpersonal barriers to care.

Selection of Sources of Evidence

**Figure 2:**
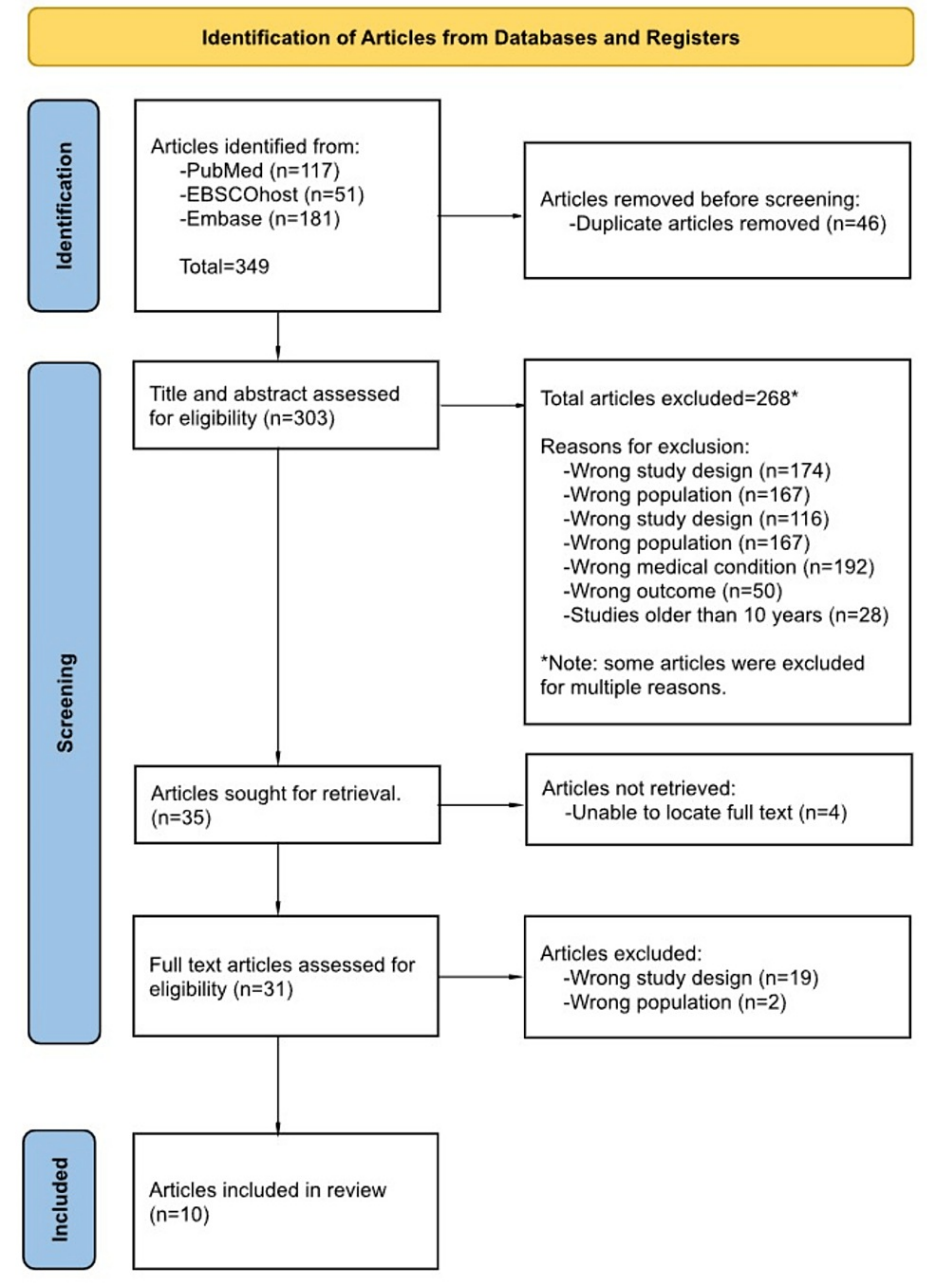
PRISMA 2020 flow chart outlining the identification and selection process used to obtain the final 10 articles used in our study. PRISMA: Preferred Reporting Items for Systematic Reviews and Meta-Analyses

The search generated 349 articles that were uploaded to Rayyan, an organizational tool used for sorting articles for review papers (Rayyan Systems, Inc., Boston, Massachusetts, United States). Following a screening and selection process, 10 final articles were selected for analysis to be included in our scoping review. The selection of articles was summarized using a PRISMA 2020 flow diagram for transparency, represented in Figure 2.

Critical Appraisal of Individual Sources of Evidence

An assessment of the quality of the 10 articles that were selected in Tier 2 was performed. In order to obtain a qualitative score for each article in terms of measures taken to prevent author bias and poor study design, each article was evaluated using the critical appraisal guidelines produced by the Joanna Briggs Institute (JBI), 2015. The checklist published by JBI was used to stratify the articles into categories to ensure that the data from all articles selected was based on statistically significant evidence with control of confounding factors. Each article was assessed by different standards in accordance with the experimental design of the study. Upon examination, six articles were found to meet 100% of the JBI criteria, one was found to meet 90%, two met 81%, and two met 75%. The threshold score determined by JBI to be classified as having a “low risk of bias” is 70%. With these findings, all of the articles that had been selected in the Tier 2 review were found to have a low risk of bias and were included in the study.

Data Charting Process

The following data were extracted from the 10 articles identified: authors, year of publication, study design, journal of population, study population, study aim, recommendations, and limitations. Key SDOHs that were referenced by the studies were recorded and listed by the number of articles that acknowledged each social determinant. The research articles were then grouped based on the recognition of each SDOH in order to synthesize the results of our study. Table 1 reports the findings of the data charting process.

**Table 1:** Summary of data extracted from included articles. UARDS: United States Renal Data System; SES: socioeconomic status; CKD: chronic kidney disease; GFR: glomerular filtration rate; ESRD: end-stage renal disease; MHS: military health system

Reference	Study Design	Data Collection	Study aim	Key Findings	Recommendations	Limitations
Zarkowsky et al. (2015) [20]	Retrospective analysis	n = 396,075	Use the US Renal Data System to determine the relationship between racial status and availability of renal dialysis care.	Black, Hispanic, and Asian patients with CKD are more likely to experience limited access to kidney specialists.	Awareness is needed in areas that can be addressed to prevent inequalities in renal dialysis care between racial demographics.	Due to the variability in practitioners who contribute data to the USRDS, there may be inconsistencies in the data entered. The providers may have had clinical reasons that accounted for delays that are not apparent when looking back retroactively.
Lee et al. (2020) [25]	Cross-sectional study	n = 2797	To investigate the inequalities in renal hemodialysis services among different ethnic and racial populations residing in the city of Chicago.	While racial and ethnic minority populations in the southern and western regions of the city were found to live in areas of lower economic income, it was found that African American patients had overall shorter travel distances, while Hispanics had access to better facilities.	The authors recommend that follow-up studies using more recent data should explore the outcomes of policy changes, community interventions, and urban transformations.	Due to the limitations on patient ZIP code data, the results from this study may not reflect the population of the rest of the United States.
Johns et al. (2014) [26]	Cohort	n = 11,027	Determine if race and SES are predictors of mortality in CKD patients receiving dialysis care in the United States.	Young adult Blacks living in low SES areas had a higher prevalence of mortality than their White counterparts when controlling for confounding variables. This effect was attenuated in higher SES neighborhoods.	To further examine variables that may impact mortality differentially among the two subgroups including residential segregation, neighborhood poverty density, racial composition of the dialysis facility patient population, patient comprehension and understanding of healthcare-related information, and patient compliance to ongoing dialysis treatment.	Limitations include not being able to examine the relations between the SES indicators fully by taking individual-level SES data into account.
Ashrafi et al. (2022) [27]	Cross-sectional study	n = 2619	Determining racial inequalities in access to healthcare among patients with CKD through four specific indicators.	CKD patients of Hispanic and Asian origin were disproportionately affected by lack of healthcare access, one of the biggest indicators being lack of mental health services.	More specific policy-based interventions that increase access to mental health services and insurance policies that don’t limit access to renal dialysis treatment.	Relatively small sample size for a cross-sectional study.
Delgado et al. (2022) [28]	Special report	N/A	In order to reevaluate and determine a means of calculating GFR while eliminating racial bias, National Kidney Foundation (NKF) and the American Society of Nephrology (ASN) convened to rethink the current method of GFR determination for detection of CKD.	The factor of race should be removed from the calculation of GFR as race is not a necessary factor in this measurement. The inclusion of cystatin C is a more reliable marker in CKD patients. The variable of race and ethnicity should be removed from all estimations of GFR across the United States.	To remove the label of race in the measurement and estimation of GFR in all patient populations. Recommendations to pursue further research in GFR estimates that are novel and eliminate racial and ethnic disparities should be fully supported, encouraged, and funded.	Limitations include the lack of research into new, clinical, biomarkers that are unbiased to race or ethnicity
Nee et al. (2016) [29]	Retrospective cohort	n = 739,537	Determine the relationship between economic status and the level of nephrology care received before initiating renal dialysis.	Low-income and racial and ethnic minority status patients had less of a probability of receiving pre-dialysis nephrology care.	Nephrology care in chronic kidney disease should be improved to focus on patients belonging to ethnic and racial minority demographics.	Cause-effect relationships cannot be determined because the study is retrospective. Data from healthcare practitioners may be subject to bias.
Plantinga et al. (2014) [30]	Ecological study	n = 5184	Determine if neighborhood poverty is associated with a lack of nephrology care prior to diagnosis with ESRD.	After a multi-level ecological study, it was determined that there was no association between location in a poverty area and barriers to receiving care from nephrology prior to ESRD among the facilities examined.	Although there are reported negative health effects of living in neighborhood poverty, the similar access to care received in pre-ESRD care throughout dialysis facilities regardless of location suggests that focused support to ease access to care in areas of high poverty is not currently needed.	This study focused on dialysis facility care prior to ESRD; however, patients might not opt to receive this care at a dialysis facility. The data of this study also concludes that renal dialysis care in lower SES areas is generally inferior. The impact of the specific location of care provided before renal failure of all patients prior to dialysis center treatment is not entirely clear. This study looked at the socioeconomic factors of the neighborhood these dialysis facilities were in and not the surrounding areas, which does not take into account the geospatial concentration of poverty.
Velez-Bermudez et.al (2022) [31]	Interview/survey	n = 40	To explore different factors that may affect patients’ decision in choosing between in-center vs. home dialysis.	Healthcare access and engagement throughout patients’ dialysis experience affected patients’ decision-making. Race, ethnicity, and language barriers also affect outcomes. Underrepresented backgrounds should be prioritized with additional resources.	Greater equity can be achieved by addressing macro-level factors.	This was a qualitative study. The conclusions were interpreted based on the participants' descriptions and impressions.
Madden and Qeadan (2017) [32]	Case-control study	n = 4104	Determine the relationship between social support and emergent dialysis among undocumented immigrants with ESRD	Interpreter support significantly increases the likelihood of receiving dialysis.	The need for inclusive pro-immigrant legislation that supports Medicare-ineligible individuals.	Chart data does not specify language ability among providers. New Mexico is geographically not representative of the entire country.
Norton et al. (2021) [33]	Cross-sectional study	n = 105,504	To assess whether there are CKD disparities present in the MHS among beneficiaries who receive universal health care.	CKD was more prevalent in beneficiaries who were Black, lower rank, and residents of lower socioeconomic income areas. However, prevalence was lower in beneficiaries who are unmarried. Despite universal health coverage, CKD disparities still exist in the MHS.	To really address the racial and socioeconomic disparities in CKD, access to universal health care alone is not enough to mitigate the problem. Rather, broader interventions addressing social risk factors are necessary.	Because this is a cross-sectional study, cause-and-effect relationships cannot be determined. More information is needed on laboratory practices and diagnostic criteria in the civilian world to accurately compare CKD prevalence. Additionally, because the MHS population is constantly moving and changing housing locations, ZIP codes may not accurately reflect household income.

Results

Following the screening process, a final total of 10 of the most relevant articles were selected utilizing the eligibility criteria. This collection of articles contained a variety of different types of study designs, including one special report, three cross-sectional studies, one survey, one case-control study, one cohort, two retrospective studies, and an ecological study. The populations of the studies we included ranged from 40 to over 800,000 participants. The collective pool of studies included data from over 2.1 million participants. The age of the studies ranged from one year to nine years, with the average being 4.6 years. The main SDOH that were discussed by the articles in this selection are listed below.

Neighborhood of Residence as an SDOH

Lee et al. (2020) investigated the impact of the racial composition of a patient’s neighborhood on their access to hemodialysis treatment facilities [25]. The study found that the neighborhoods with the longest commute time to dialysis facilities were predominantly Hispanic, while the neighborhoods with the best access to dialysis facilities were either predominantly Black or mixed communities. They noted that the process of gentrification can have both positive and negative effects on access to care.

Johns et al. (2014) examined the effects of neighborhood composition in renal dialysis patients [26]. The study points out that after controlling for age and sex, African American CDK patients have a lower life expectancy than what would otherwise be expected compared to White CKD patients, and this relationship is dependent on neighborhood SES. These findings may be attributable to hemodialysis facility access and health insurance access. Patients from other racial demographics did not experience the same decrease in life expectancy even if socioeconomic factors were controlled in both populations. 

Mental Healthcare Access as an SDOH

While other studies focused mainly on access to hemodialysis care in CKD patients, Ashrafi et al. (2022) demonstrated that mental health may pose a barrier to treatment in CKD [27]. Although mental health access and nephrology care access are two separate issues, mental health can be viewed as an SDOH that may in itself impact a patient's access to nephrology care. The study found that while over nine out of 10 patients of all racial demographics lacked mental health care access, the racial demographic most likely to lack mental healthcare access was Asian patients. They found that Hispanic CKD patients were the most lacking in overall healthcare access. Improvement in mental health access may improve patient compliance and adherence to treatment plans.

GFR Methodology in Medicine as an SDOH

As a calculated measure used as a component of health care services, GFR methodology can be viewed as an SDOH because it influences access to care. A special report by Delgado et al. (2022) discusses the formation of a special council formed by national kidney organizations with the objective of reassessing the equations used to determine objective markers of kidney function [28]. The previously accepted equations for determining kidney function included the social construct of race as a variable. This may have delayed the diagnosis of CKD and therefore created barriers to dialysis and transplant access for African Americans. Patients may experience disparities depending on which physicians they see, and the type of equation that those physicians prefer to use. Over the course of more than 40 meetings, the council brainstormed a total of 26 alternative methods to measure kidney function by GFR while avoiding the creation of such disparities. The final consensus by the council is that the most effective method of GFR estimation that is racially unbiased is the removal of the race variable from the equation. Further recommendations from this report included widespread adoption of the estimation of renal function by use of Cystatin C as a biomarker, as well as future research to identify other potential biomarkers. Because race is not a variable in the equations that utilize Cystatin C, these equations are less likely to contribute to racial disparities, and they are also regarded to be more accurate [28].

SES as an SDOH

While there was some overlap, two articles were identified that mainly addressed SES as a social determinant of healthcare access in chronic kidney disease. Nee et al. (2016) examined access to renal hemodialysis care among racial minority patients affected by poverty, as determined by zip code [29]. The study found that patients who were affected by poverty were much less likely to have access to care, especially those belonging to African American and Hispanic demographics. They point out that this disparity may compound and increase the rate of CKD progression that African American patients experience compared to White patients. Plantinga et al. (2014) investigated the relationship between patients from neighborhoods of lower socioeconomic status and access to renal hemodialysis facilities [30]. The study found that many dialysis facilities are located in low socioeconomic neighborhoods, and they did not find any barriers to renal dialysis access for patients from these neighborhoods. The study concluded that patients from low SES neighborhoods already have adequate access to renal hemodialysis. However, patients from these neighborhoods may have a smaller selection of facilities to choose from than patients from higher socioeconomic areas.

Primary Language Spoken as an SDOH

Even if a CKD patient is physically able to get to a treatment facility, they will encounter a barrier to accessing care if they are unable to communicate with the facility staff. Along with disparities in healthcare access among different racial demographics, Velez-Bermudez et al. (2022) examined the impact of language barriers in CKD patients undergoing hemodialysis treatment through patient interviews [31]. Spanish and other non-English speaking patients reported a lack of access to translation services in dialysis facilities, which created a barrier to their comprehension of the treatment process and options. In addition to information about their treatment, Spanish-speaking patients are also prone to missing important information about legal medical documentation and may be asked to sign paperwork that has not been translated into their language [31]. The article pointed out that hospital visitor restrictions imposed by the global COVID-19 pandemic in 2019 inhibited patients with language barriers from bringing family members with them to dialysis appointments to serve as translators, highlighting a potential contribution to barriers of care [31]. 

Immigration Status as an SDOH

In addition to language barriers, CKD patients from countries outside the United States may experience barriers to dialysis healthcare access due to their immigration status. Madden and Qeadan (2017) report that Hispanic undocumented immigrant patients are often barred access to health insurance coverage, and often present to the Emergency Department for dialysis treatment rather than going to a dialysis treatment facility [32]. The study found that undocumented immigrant Hispanic patients are even more at risk of seeking emergency dialysis care if they also have a concurrent language barrier. Because these patients wait until their situation becomes an emergency before seeking treatment, they do not receive the proper nephrology care needed to manage the progression of their CKD. The Hispanic population has twice the likelihood of the White population of developing CKD in their lifetime, so these additional barriers to treatment can further worsen the already poor prognosis in this demographic [32]. The authors state that further research is required to investigate whether immigrants of other ethnicities experience similar barriers to renal dialysis access [32].

Military Rank as an SDOH

Norton et al. (2021) investigated disparities in CKD within the context of the military healthcare system. The results of their cross-sectional study suggest that there is an increased incidence of CKD in older enlisted service members as compared to senior officers [33]. Because the military provides all its members with adequate insurance coverage through the TRICARE Health Plan, this disparity is unlikely to be caused by differences in insurance coverage. The authors conclude that the findings of their study may be attributable to social and cultural factors [33].

Discussion

From the thesis described in the introduction, some of the questions presented at the beginning of the study have been investigated and answered, while others still represent gaps in the literature that future studies should address. Topics that were well covered included SES, language barriers, and immigration status. While Nee et al. (2016) supported the hypothesis that lower SES communities have less access to care, it conflicted with the findings from Plantinga et al. (2014), which reported that many low-income communities have high enough numbers of renal dialysis facilities to ensure adequate access to care [29,30]. It may be that SES might be more of a barrier to access in some locations than others, which may vary by geographic location. A lack of proper pre-dialysis care may promote the demand for access to dialysis in these communities.

The studies reviewed supported the fact that both immigration status and language are social determinants of renal care access and suggest that they have significant overlap [30,31]. However, there were many social determinants that were not addressed in the articles that met the study inclusion criteria. The topics of levels of education, incarceration status, limitations in personal or nonemergency medical transportation, and changes in healthcare delivery brought on by the COVID-19 pandemic were not covered by these studies, so the research questions on those topics as SDOH remain unanswered by this review. 

This study presents implications for future research and guidelines to be used in practice. It is recommended that dialysis centers make note of the potential for language barriers to create disparities in access to care. Possible interventions include keeping multiple translations of medical paperwork and documentation on hand, as well as the utilization of virtual medical translation services. Virtual medical translation services can provide translation for patients who do not speak English in areas where in-person translators are not available. They can help to improve patient understanding and decrease medical errors through miscommunication. Additionally, eliminating the variable of race from GFR calculations and adopting equations that use Cystatin C as a biomarker may help to prevent inadvertently delaying the diagnosis of kidney disease. Future studies should be conducted to investigate a broader scope of SDOH that may impact access to renal dialysis care. Further studies should also investigate SES as a social determinant in varying geographic locations, as some regions may have better dialysis center access in low socioeconomic neighborhoods than others.

Strengths and Limitations

The articles selected for this study were found to be reliable in the quality assessment that was conducted using the guidelines published by the JBI. Therefore, there is a high level of confidence in the accuracy of the studies that addressed the review questions. However, there are several limitations to the study that must be discussed. While the study mostly identified articles that focused on African American and Hispanic populations, many studies did not focus on other racial minority groups, such as Asians, Native Americans, or Pacific Islanders. Furthermore, the categorization of racial groups can be problematic to define, over-encompassing, and may obscure disparities found in smaller groups that are often lumped into larger categories, such as the Hmong population being a sub-demographic of Asians. In addition, the study only examined articles focusing on adult populations. While kidney disease requiring dialysis treatment is more common in adults, there may be similar disparities in the access to dialysis care found in pediatrics. The study only included peer-reviewed articles published in large databases, which may exclude information from articles whose findings were not significant enough to warrant publication. 

## Conclusions

Neighborhood of residence, mental healthcare access, GFR methodology, SES, language barriers, immigration status, and military rank were identified by this study as being major SDOHs discussed by existing literature as impacting access to renal dialysis care. The aim of this study was to identify existing literature on social factors contributing to disparities in access to renal dialysis care for racial minorities in the United States, and while the study identified articles discussing these social determinants, future research is needed to determine if other social determinants of health exist that contribute to disparities in this setting. In the identification of these contributing social factors, the hope is that future action can be taken to reduce disparities in kidney health across all demographics. Because CKD is a debilitating condition for millions of people in the United States, even small improvements to healthcare access have the potential to greatly improve the quality of life for countless underserved patients. Going forward, efforts to eliminate language barriers in dialysis clinics, improve mental healthcare, and eliminate the social construct of race as a variable within GFR equations are important steps to take to reduce future health disparities, especially those related to CKD.
